# Ethnic Differences Shape the Alpha but Not Beta Diversity of Gut Microbiota from School Children in the Absence of Environmental Differences

**DOI:** 10.3390/microorganisms8020254

**Published:** 2020-02-14

**Authors:** Ke Liu, Yongling Zhang, Qinglin Li, Huan Li, Danfeng Long, Shijuan Yan, Wenjie Huang, Ruijun Long, Xiaodan Huang

**Affiliations:** 1 School of Public Health, Lanzhou University, No. 222 TianshuiNanlu, Lanzhou 730000, China; liuk17@lzu.edu.cn (K.L.); ylzhang2017@lzu.edu.cn (Y.Z.); liql16@lzu.edu.cn (Q.L.); lihuan@lzu.edu.cn (H.L.); Longdf@lzu.edu.cn (D.L.); shijuan@agrogene.ac.cn (S.Y.); 2 Agro-biological Gene Research Center, Guangzhou Academy of Agricultural Sciences, Tianhe Distinct, Guangzhou 510640, China; huangwenjie@agrogene.ac.cn; 3 School of Life Science, Lanzhou University, No. 222 TianshuiNanlu, Lanzhou 730000, China

**Keywords:** Qinghai–Tibetan Plateau, dietary habit, ethnicity, gut microbial diversity, Miseq

## Abstract

Although the human gut microbiome is shaped by factors such as diet, environment, and genetic background, most studies investigating the relationship between ethnicity and microbiota have compared groups living in separate geographical locations. To isolate the effects of ethnicity on microbial diversity by minimizing environmental differences, we selected 143 school children from Han, Tibetan, and Hui populations from the same town on the Qinghai–Tibetan Plateau for fecal microbiome 16S rDNA sequencing. We characterized the diversity, identified signature taxa, and performed correlation analysis between diet and community composition. Firmicutes (47.61%) and Bacteroidetes (38.05%) were dominant phyla among the three ethnic groups; seven genera showed significant differences in relative abundance. Tibetan populations had a higher relative abundance of *Oscillibacter* and *Barnesiella*, compared with Han and Hui populations. Alpha diversity analyses (observed species, ACE, and Shannon indices) showed that the Tibetan population had the highest diversity compared to the Hui and Han groups, whereas beta diversity analysis revealed no significant differences between groups. The consumption of grains, milk, eggs, and fruits were positively correlated with specific taxa. Under similar environments and diet, ethnic background significantly contributed to differences in alpha diversity but not beta diversity of gut microbiota.

## 1. Introduction

The human body is populated by trillions of microorganisms, which encode 100-fold more unique genes than our own genome [1]. In particular, the gastrointestinal tract harbors the largest number and greatest variety of microbes compared with other organs. The gastrointestinal microbiota performs numerous physiological functions, including nutrient metabolism, immunomodulation, and maintenance of the gut barrier [2,3,4]. Gut microbiota therefore play an important role in maintaining human health. Previous reports have proposed that an imbalance in the community composition of gut microbiota may be associated with the development of diseases such as inflammatory bowel disease, obesity, allergies, and psychological disorders [5,6,7]. Understanding the factors that adversely affect the composition of gut microbiota provides a robust means of maintaining a stable, beneficial microbial community, and can thus mitigate the risk of diseases related to the intestinal microbiome. Many of these factors, such as geography and environment, age, diet, and lifestyle, have been previously examined for their contribution to shaping the structure of the human gut microbiota [8,9,10,11,12]. Similarly, metagenomic analysis by Zhernakova et al. [13] revealed that diet, age, and sex were correlated with microbiota composition. In another study, 16S rDNA sequencing of gut microbiota of 2084 participants from six countries showed that differences in ethnicity explained inter-individual dissimilarities in microbiota composition [14].

The Qinghai–Tibetan Plateau, the world’s largest and highest plateau, is often called “the roof of the world” and “the world’s third pole.” This region is described as one of the most extreme living environments, characterized by low oxygen, low air pressure, low temperature, and high radiation [15,16]. However, indigenous people in this region, consisting of Tibetan and Han majority ethnic groups, along with other minority groups, have adapted to the harsh climate. Due to the geographic separation, residents of this region have developed a genetic background distinct from other members of their respective ethnic groups in more accessible regions, as well as distinct cultures, and dietary habits. In addition, the large Tibetan population and close proximity of several ethnic groups make the Qinghai–Tibetan Plateau an attractive model for understanding the variation among gut microbiota and for investigating the factors affecting diversity among intestinal microbiota.

Several previous studies in the Qinghai–Tibetan Plateau examined altitude and ethnicity as strong determinants of gut microbiota in comparisons of Tibetan and Han populations. For example, Li et al. (2016) [17] found that the Tibetan population had a higher abundance of *Prevotella,* whereas the Han population was enriched with *Bacteroides*, and that these taxa were correlated with altitude and genetic background, thereby implicating these factors in the structural dynamics of intestinal microbiome. Li et al. (2015) [15] also suggested that altitude, ethnicity, and dietary factors may explain the differences in microbiota between Tibetan and Chinese Han. However, most of these studies examined human subjects living in geographically distant areas, potentially introducing region-specific variability such as differences in climate and air pollution, or a wider variety of available foods, which cannot be excluded as contributing factors to the composition of gut microbiota. In light of these possible confounding factors associated with geography, we sought to isolate the factors of ethnicity (genetic and cultural background) and dietary intake to investigate their role in shaping microbiota.

Age has also been shown to contribute to microbiota composition, and many studies have examined the development of gut microbiota during infancy because early microbial colonization is crucial for infant health and may also impact health in later life [18,19]. However, relatively little information is available on the gut microbiota of school children, which is surprising given that intestinal microbiota at this developmental stage are comparatively unstable and susceptible to dysbiosis induced by environmental stress, poor diet, and antibiotic use [20]. For example, both undernourished and obese school children are reported to have lower bacterial diversity and richness than normal-weight children [21].

In this study, we sought to determine if ethnicity and diet were drivers of microbiota composition and diversity in children in the absence of environmental differences such as altitude, climate, wide variation in food sources, pollution, and economic disparity. We thus compared the community structure of gut microbiota of 143 school children from three ethnic backgrounds, Tibetan, Han, and Hui, in the Qinghai–Tibetan Plateau. We recruited subjects who lived in the same town, from families with similar economic conditions. In these subjects, we compared differences in relative abundance of specific taxa, as well as differences in alpha and beta diversity of their gut microbiota, identified signature taxa associated with each group, and identified taxa that were correlated with specific foods. This work provides insight into the impact of genetic and cultural background and specific dietary components on the diversity and distribution of microbiota found in school children from three distinct ethnic groups living in close proximity.

## 2. Materials and Methods

### 2.1. Ethic Approval and Consent to Participate

All procedures performed were approved by the Medical Ethics Committee of school of public health in Lanzhou University (No. 20170227-1, 27 February 2017). Participants received detailed information about the study and informed consent was obtained from by guardian and the administrator/teacher of Labrang Primary School.

### 2.2. Study Site and Sampling

A total of 143 healthy school children between the third and fifth grades were recruited for sampling. All participants were from the Labrang Primary School in Xiahe County of the Gannan Tibetan Autonomous Prefecture located in the Qinghai–Tibetan Plateau (long. 102°31′25″, lat. 35°12′9″). Information on anthropometric measurements, dietary habits, and family background was collected from each subject through the school’s admission record and questionnaire survey. Anthropometric measurements, including height and weight, were performed by undergraduate students from Lanzhou University. The height and weight of subjects were measured with a mechanical altimeter and the RGZ-120 electronic weight scale (Suhong Co. Ltd., Changzhou, China), respectively. The reading was maintained at one decimal. The body mass index (BMI) was calculated from weight in kilograms divided by height in meters squared. The children did not take any antibiotics within the last three months before sampling, and their age, sex, dietary habits, and ethnic background were recorded. Sample collection was conducted on the morning of 21 September 2017. Fresh stool samples were maintained in liquid nitrogen immediately after collection, then, the samples were stored at −70 °C for further analysis.

### 2.3. DNA Extraction

Fecal DNA was extracted using a QIAamp DNA Stool Mini Kit (QIAGEN, Hilden, Germany) according to the manufacturer’s instructions. The DNA concentration was determined using a Nanodrop ND-2000 spectrophotometer (Thermo Fisher Scientific, Waltham, MA, USA). The integrity and size of the DNA were checked by 1% agarose gel electrophoresis. All DNA samples were stored at −20 °C until further processing.

### 2.4. Polymerase Chain Reaction (PCR) Amplification and High-Throughput Sequencing

Bacterial 16S rDNA amplification and library construction were performed according to the 16S Metagenomic Sequencing Library Preparation guide by Illumina. The variable V3–V4 region of the bacterial 16S rDNA gene from each DNA sample was amplified using the primer F (CCTACGGGNGGCWGCAG) and primer R (GACTACHVGGGTATCTAATCC). PCR was performed on the ABI 2720 Thermal Cycler (Thermo Fisher Scientific, USA) using 25 µL reaction volume containing 2.5 µL of microbial DNA (5 ng/µL), 5 µL of Amplicon PCR Forward Primer 1 (µM), 5 µL of Amplicon PCR Reverse Primer (1 µM), and 12.5 µL of 2x KAPA HiFi HotStart ReadyMix (KAPA Biosystems, Wilmington, MA). The cycling parameters were as follows: 95 °C for 3 min, followed by 25 cycles of 95 °C for 30 s, 55 °C for 30 s, 72 °C for 30 s, and a final extension at 72 °C for 5 min. The final PCR products were quantified with an Agilent 2100 bioanalyzer (Agilent Technologies, Palo Alto, CA, USA) and purified with AMPure XP beads (Beckman Coulter, Pasadena, CA, USA), according to the manufacturer’s protocol. Sequencing was conducted with MiSeq Reagent Kit v3 (Illumina, San Diego, CA, USA). The raw reads were deposited by accession number PRJEB30788 into the European Nucleotide Archive (http://www.ebi.ac.uk/ena/data/view/PRJEB30788).

### 2.5. Data Processing and Bioinformatic Analyses

To obtain high-quality sequencing data to improve the accuracy of subsequent bioinformatic analyses, quality control, and filtering of the raw sequence data were required. The following criteria were applied: (1) TrimGalore (version 0.4.2) software filtered out the bases with quality scores below 20 at the end of sequences, which might comprise adapter and short sequences less than 100 bp in length. (2) The paired-end sequences were merged with FLASH2 software [22], and then low-quality sequences were removed. (3) The primers in the sequences were found and removed using Mothur (version 1.41.1). (4) Sequences with less than 100 bp in length or error rate of total bases more than 2 were discarded by USEARCH (version 10.0). The high-quality sequences were clustered into operational taxonomic units (OTUs) at a 97% sequence similarity, and their representative sequences were classified using the Ribosomal Database Project [23]. Mothur was used to calculate the alpha diversity parameters, including ACE, observed species, and Shannon index, and then to discriminate significantly different species between the two groups. To assess the beta diversity, principal coordinate analysis (PCoA) based on unweighted and weighted UniFrac distances was performed. VENN analyses were also conducted using R (version 3.2.1). In addition, the rarefaction curves [24] based on the observed species at OTU level were calculated. Linear discriminant analysis effect size (LEfSe) [25] was used to screen species that can most likely to explain the differences among the Han, Hui, and Tibetan groups.

### 2.6. Evaluation of Dietary Nutrition

A food frequency questionnaire was designed to collect the dietary habits of local school children after preliminary data collection [26,27]. Food categories included grains, vegetables, fruits, poultry meat, livestock meat, seafood, dairy, beans, eggs, nuts, condiments, and sweets. The dietary habits of the school children from the three ethnic groups were studied using the prepared questionnaire. The dietary questionnaire consisted of three parts: one part was answered by the subjects, and the other two parts by their respective guardians. The dietary frequency questionnaires were adopted to obtain the intake of each food that every child had in the past six months. CDGSS3.0 nutrition software was used to analyze the nutritional intake of each subject. Observed species and Shannon indexes were used to assess the alpha diversity of diets, while principal component analysis (PCA) was conducted to evaluate the beta diversity of food components in the diets of each ethnic group.

### 2.7. Statistical Analysis

One-way analysis of variance (ANOVA) was used to evaluate the differences in relative abundances of taxonomic units across different groups, with P values adjusted using the Bonferroni correction. Comparison of the alpha diversity indices among groups was tested using the Kruskal–Wallis test. *p* values were corrected using the false discovery rate control. Pearson’s correlation analysis evaluated the linkages between gut microbial structure and environmental factors (diet and antibiotic residues) by using the R package. Permutational multivariate analysis of variance (PERMANOVA) was performed using the R package vegan to evaluate whether gut microbiota structure was significantly different based on the UniFrac distances with 9999 permutations [28]. Statistical analyses were performed using SPSS version 22.0.

## 3. Results

### 3.1. Baseline Characteristics of the Subjects in the Three Ethnicity Groups

A total of 50 Han, 49 Hui, and 44 Tibetan children aged 7–12 (9.34 ± 0.99) years old from Gannan Tibetan Autonomous Prefecture in the Qinghai–Tibetan Plateau were included in this study. The BMI of the participants ranged from 12.10 to 25.76 (16.05 ± 2.62), and the proportion of males was 50.3%. The proportion of sex, BMI, and age showed no significant difference among different groups (*p* > 0.05).

### 3.2. DNA Sequencing and Filtering

A total of 11,791,079 raw reads were obtained by high-throughput sequencing of the V3–V4 region of the 16S rDNA gene of bacteria and archaea from 143 fecal samples. After filtering low-quality reads, 9,876,756 clean reads were generated with a median read length of 414 bp, and nearly 19.23% of raw data were filtered. The average number of high-quality reads per sample reached 69,068 and ranged from 45,140 to 111,335 across all samples.

### 3.3. Bacterial Compositions of Fecal Samples

All 9,876,756 high-quality sequences were clustered into OTUs at the 97% similarity level. A total of 1265 OTUs were identified. Across all samples, nearly 99.94% of sequences were assigned to a bacterial kingdom, whereas a few reads remained unclassified.

**Figure 1:**
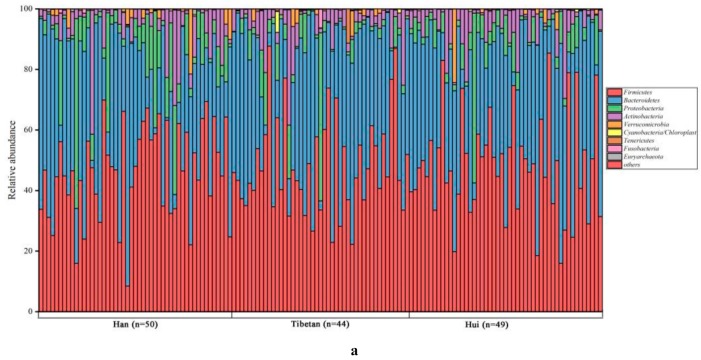

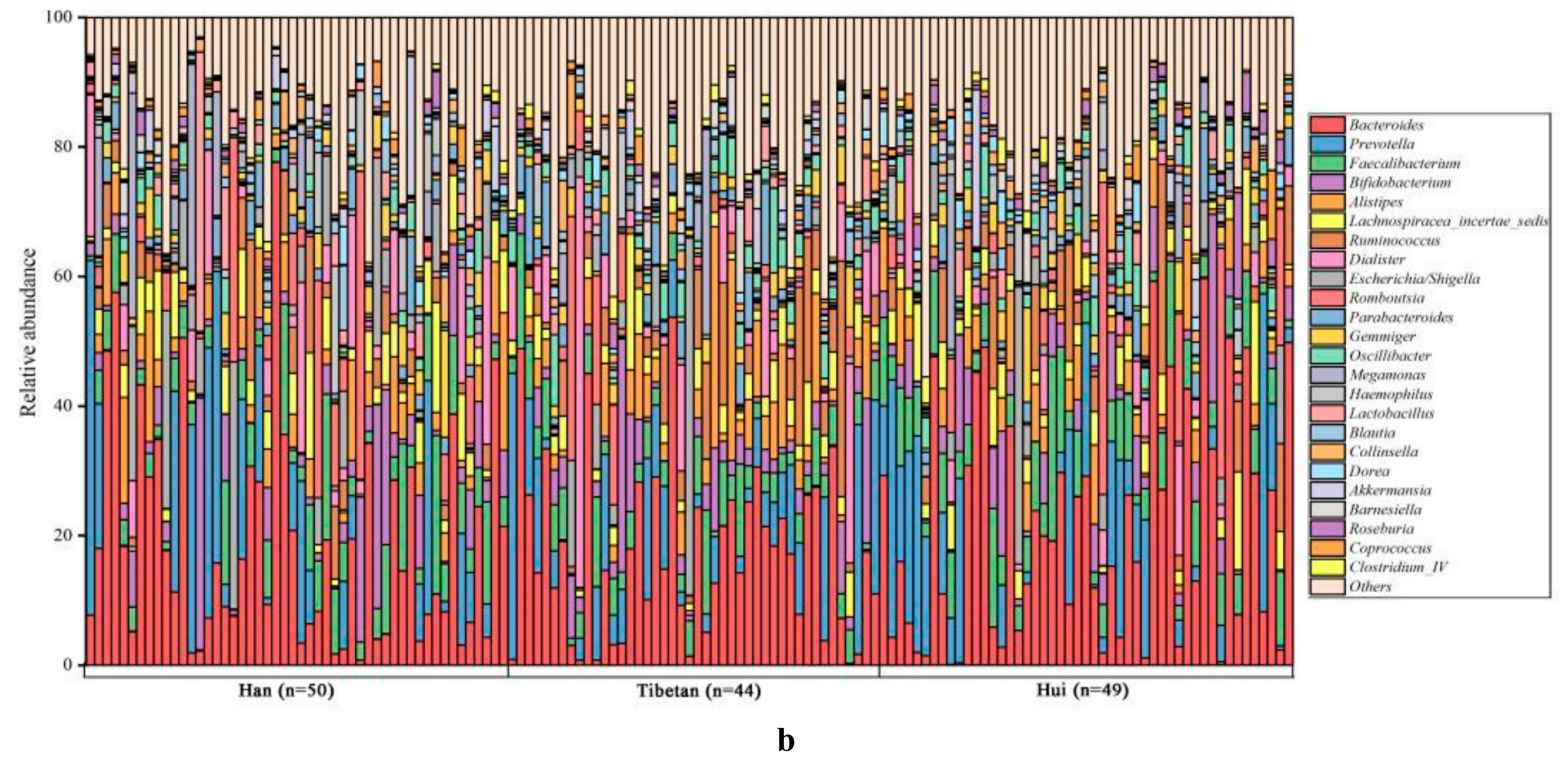
Relative abundance of bacterial phyla (**a**) and genera (**b**) in the fecal microbiota of all participants.

**Figure 2:**
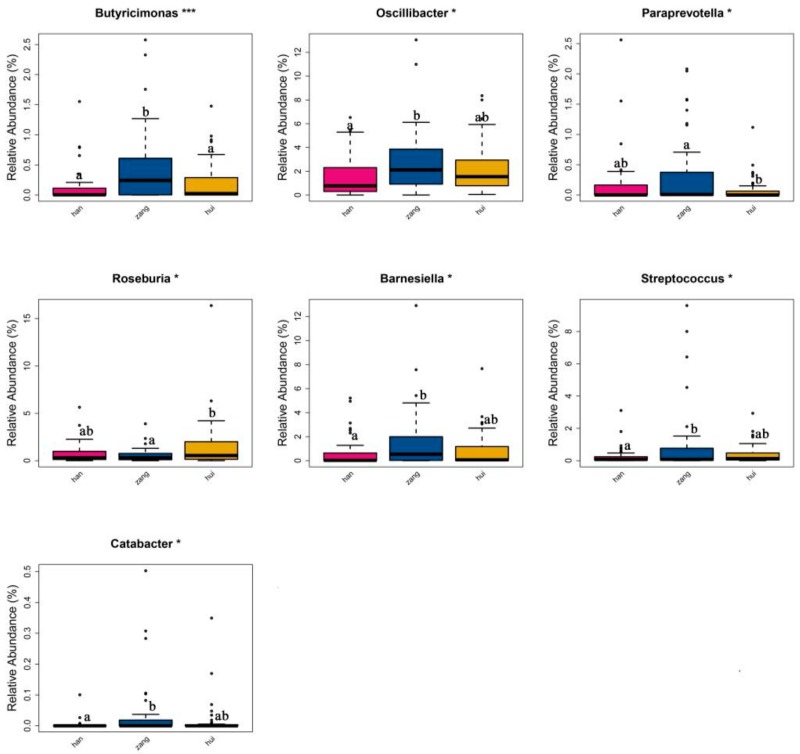
Box plots of the relative abundance of significantly different genera in the Han, Tibetan, and Hui populations. Boxes represent the interquartile range, and the center line indicates the median. Whiskers denote the lowest and highest values (ANOVA). ^a,b^ indicate significant differences between groups.

The overall gut microbiota composition of all participants at the phylum and genus levels is shown in Figure 1. A total of 25 bacterial phyla and 253 genera were detected from the samples. The dominant phyla among all groups were Firmicutes (mean relative abundance = 47.61%), and Bacteroidetes (38.05%), followed by Proteobacteria (6.80%), and Actinobacteria (6.12%). At the genus level, *Bacteroides* (19.41%) predominated, followed by *Prevotella* (8.31%), *Faecalibacterium* (6.52%), and *Bifidobacterium* (4.79%). Examination of the mean relative abundances of individual phyla within each group revealed that Firmicutes was the most predominant phylum, contributing 48.34%, 46.25%, and 48.36% of the fecal microbiota in Tibetan, Han, and Hui groups, followed by Bacteroidetes, contributing 39.19%, 36.27%, and 38.88%, respectively. In agreement with the similarity of these proportions, ANOVA showed no statistical significance (*p* > 0.05) in the microbiota composition between ethnic groups. To further characterize the differences in taxonomic representation, we examined genus-level taxa using ANOVA and identified seven genera for which the relative abundances were significantly different among the three ethnic groups (Figure 2). The mean relative abundances of *Butyricimonas*, *Oscillibacter*, *Barnesiella*, and *Catabacter* were higher in Tibetan populations than in Han populations (*p* = 0.001–0.033), whereas *Streptococcus* showed the opposite distribution (*p* = 0.034). Compared with that of the Tibetan group, the gut microbiota of the Hui group was significantly enriched for *Roseburia* (*p* = 0.029), whereas *Butyricimonas* and *Paraprevotella* were lower in Hui populations than in Tibetan (*p* = 0.013–0.014).

### 3.4. Alpha Diversity of Gut Microbes from the Three Ethnic Groups

**Figure 3:**
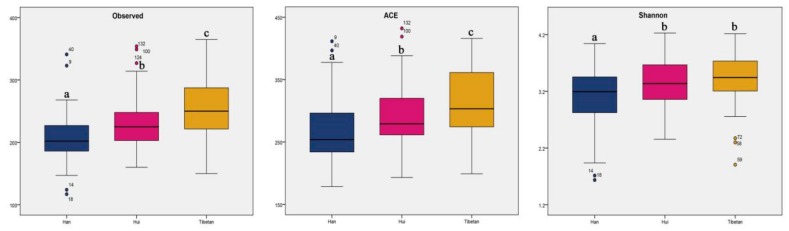
Box plots of alpha diversity in Han (blue), Hui (red), and Tibetan (yellow) ethnic groups on the basis of the number of observed species, ACE, and Shannon index. ^a,b,c^ indicate significant differences between groups.

The rarefaction curves of all samples almost reached a plateau, suggesting that the sequencing depth was sufficiently even, although additional sequencing may acquire some additional rare bacterial species (Figure S1). Differences in species representation (richness) and diversity (richness and abundance) of fecal microbial communities within ethnic groups were assessed for all of the 143 subjects using three indices: ACE, observed species, and Shannon index (Figure 3). The observed species and ACE indices revealed significant differences in diversity within groups, which exhibited a decreasing trend in species richness from the Tibetan to Hui and then Han groups (Kruskal–Wallis test, *p* < 0.05). The Shannon index was also lower in the Han group than in the Hui and Tibetan groups (Kruskal–Wallis test, *p* < 0.05), although no significant differences were observed in the Tibetan and Hui groups.

### 3.5. Beta Diversity of Gut Microbiota among the Three Ethnic Groups

**Figure 4:**
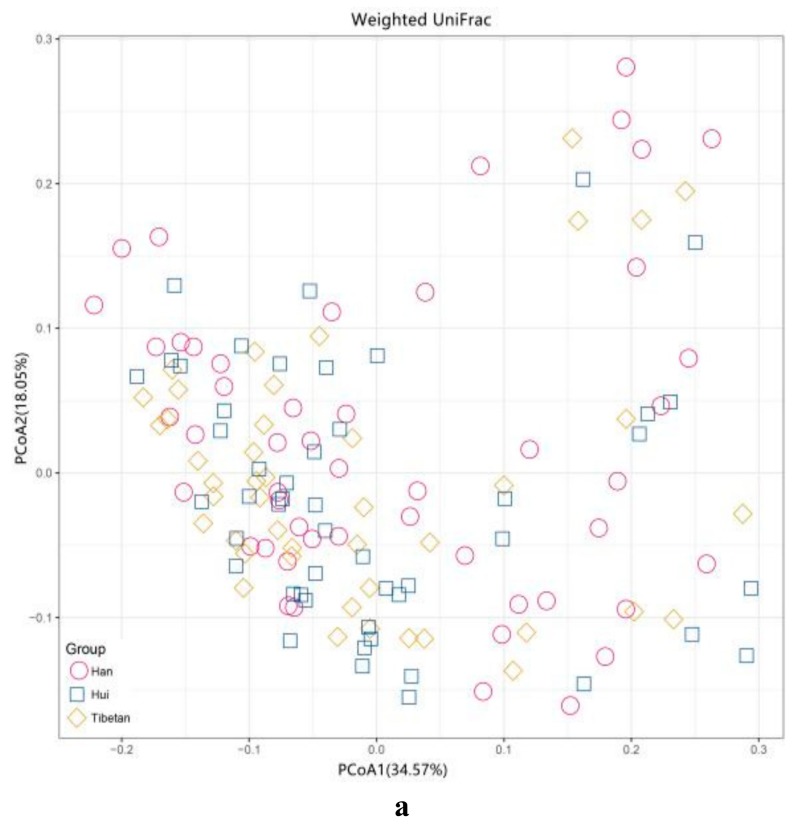

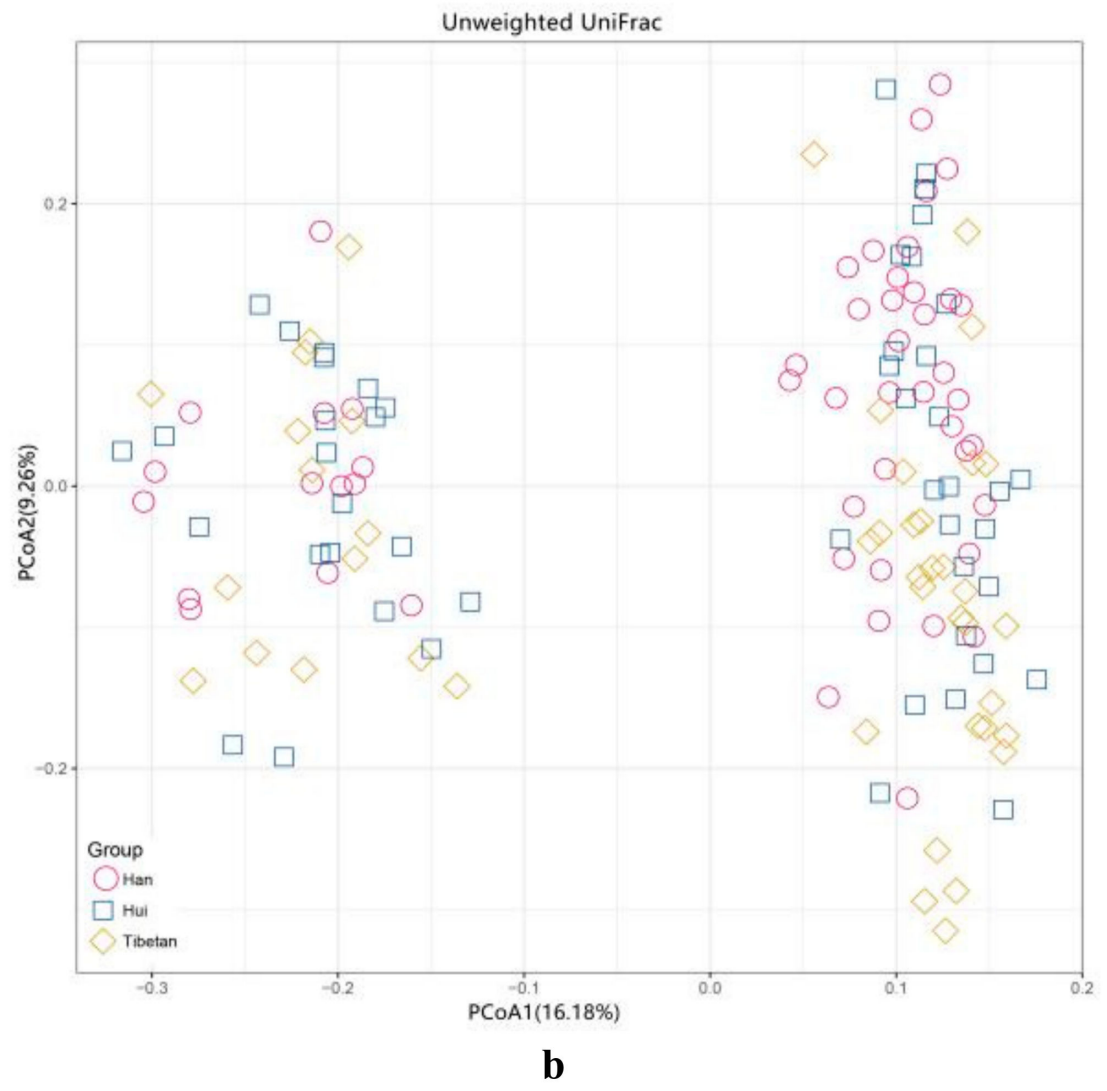
Principal coordinate analysis (PCoA) derived from (**a**) weighted and (**b**) unweighted UniFrac distances among samples of the three ethnic groups. The percent of variation explained by each axis is shown in square brackets, with Han samples represented by purple circles, Hui as blue squares, and Tibetan as yellow squares.

Principal coordinate analysis (PCoA) based on weighted and unweighted UniFrac distances was performed to uncover differences in the structure of gut microbiota across all samples based on the relative abundance of OTUs. The results revealed only slight variation (Figure 4). Similarly, PERMANOVA did not show any significant differences distinguishing the three ethnic groups from the whole (*p* < 0.05).

### 3.6. Microbial Signatures in Different Samples

**Figure 5:**
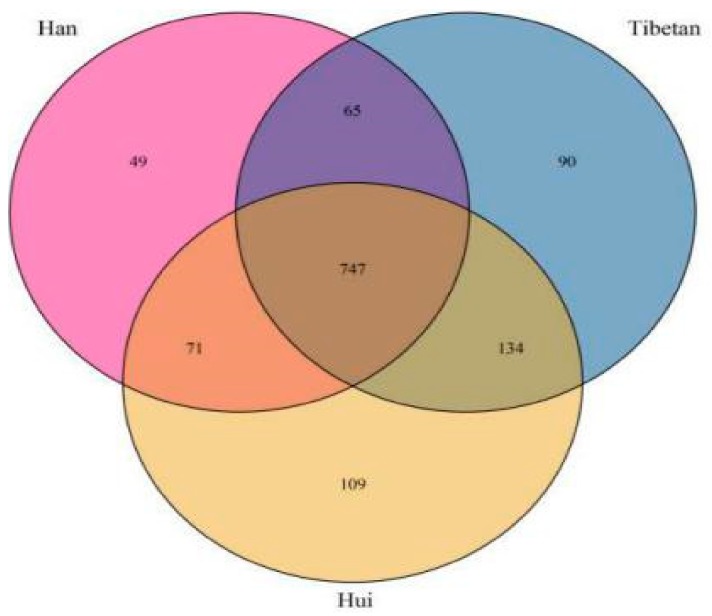
VENN analyses among the three ethnic groups.

VENN analyses showed that 747 of the detected OTUs were shared by all samples, while other OTUs were unique to the three ethnic groups, including 90 OTUs in the Tibetan group, 49 in the Han group, and 109 in the Hui group (Figure 5). LEfSe was used to identify the specific taxa that were variably distributed in the three ethnic groups. Signature gut microbiota included *Methanobacteriaceae*, *Veillonellaceae*, and Gammaproteobacteria in the Han samples and *Porphyromonadaceae*, *Catabacteriaceae*, *Christensenellaceae*, *Bdellovibrionaceae*, and *Anaeroplasmataceae* in the Tibetan samples. Some *Bacteroidetes incertae sedis* were found uniquely in the Hui samples (Figure S2).

### 3.7. Correlation Analysis between Bacterial Genera and Diet

**Figure 6:**
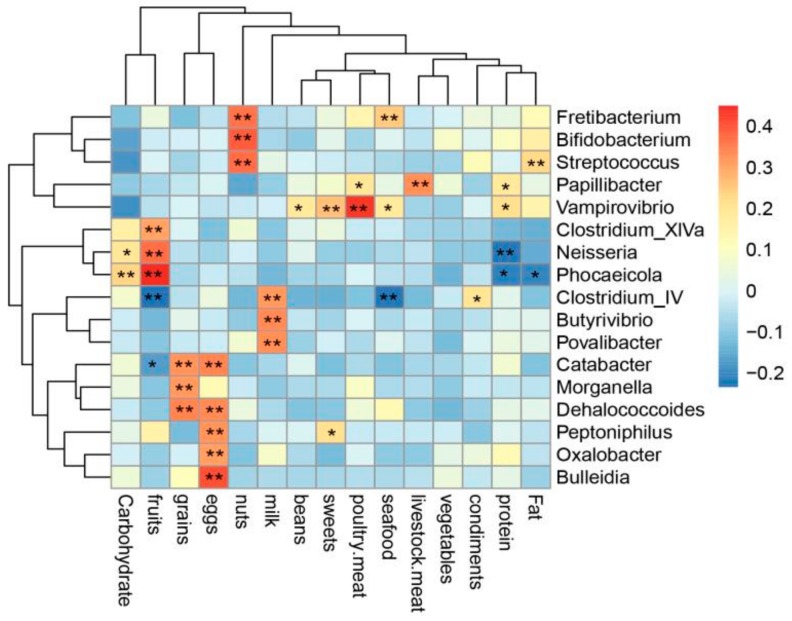
Correlation of genus with dietary intake (* *p* < 0.05, ** *p* < 0.01; *p* value was calculated using Pearson’s correlation).

The correlation analysis between genus-level taxa and diet in different ethnic groups is shown in Figure 6. The correlation coefficients above 0.3 are displayed in the figure. *Oxalobacter*, *Catabacter*, *Bulleidia*, *Peptoniphilus*, and *Dehalococcoides* were positively correlated with egg intake (r = 0.310–0.406, *p* < 0.01). Furthermore, fruit intake was potentially associated with the relative abundance of *Clostridium*_XlVa, *Neisseria*, and *Phocaeicola* (r = 0.301–0.450, *p* < 0.01). *Bifidobacterium*, *Streptococcus*, and *Fretibacterium* were positively correlated with the consumption of nuts (r = 0.61–0.394, *p* < 0.01). The relative abundance of some genera was correlated with specific meats. Specifically, poultry consumption was significantly linked to the relative abundance of *Vampirovibrio* (r = 0.434, *p* < 0.01); consumption of livestock meat was positively associated with the relative abundance of *Papillibacter* (r = 0.327, *p* < 0.01). In addition, grain intake showed a positive correlation with three genera (*Catabacter*, *Morganella*, and *Dehalococcoides*), and milk intake was significantly associated with three other genera (*Clostridium_IV*, *Butyrivibrio*, and *Povalibacter*). However, energy from proteins, fats, and carbohydrates had no significant correlation with gut microbiota.

### 3.8. Change of Dietary Habits among the Three Ethnic Groups

The average daily intake of 12 specific foods for all participants is presented in Table 1. Although dietary patterns differed slightly among the three ethnic groups, grains, fruits, and vegetables comprised the main sources of food. Consumption of vegetables, seafood, and fruits was significantly lower in the Hui subjects than in the Han subjects (*p* < 0.01), while the consumption of poultry meat in the Hui population was significantly higher than in the Tibetan population (*p* < 0.05). Total energy and the energy ratio from macronutrients (proteins, carbohydrates, and fats) are shown in Table 2. Carbohydrates provided the predominant source of energy, followed by fats, and proteins. Total energy and the percentages of energy from proteins and carbohydrates did not differ among the three ethnic groups, but the proportion of energy from fats was higher in the Tibetan group than in the Hui group (*p* < 0.01). Through alpha diversity analysis, using the observed species and Shannon diversity indexes for diet, we found no significant differences across ethnicities (*p* > 0.05) (Figure S3). For beta diversity analysis, PCA showed that the distribution of the diets largely overlapped. PERMANOVA analysis confirmed that dietary structure was also not significantly different across ethnicities (*p* > 0.05) (Figure S4).

## 4. Discussion

We studied the composition of gut microbiota from Tibetan, Han, and Hui children from similar economic strata, residing in the same town in the Qinghai–Tibetan Plateau. The subjects were all within the 7–12-year-old age range, and had BMIs ranging from 12.10–25.75. We also collected information regarding the dietary nutrition of subjects to determine if there was any relationship between differences in nutrient intake and microbial diversity in the gut.

Our data showed that Firmicutes and Bacteroidetes dominated these bacterial communities, in agreement with other studies on gut microbiota. Although Schnorr et al. [29] and Zhang et al. [30] reported a higher relative abundance of Firmicutes (>70%) in the human gut, a previous study of gut microbes in Tibetans revealed a higher abundance of Bacteroidetes (60.00%) and fewer Firmicutes (29.04%) [31]. In the present study, Firmicutes (47.61%), Bacteroidetes (38.05%), and Proteobacteria (6.80%) represented 92.46% of the sequences. The relative proportions of Firmicutes and Bacteroidetes are reportedly influenced by many factors [32,33]. The intestinal microbes and host form a symbiotic, long-term relationship early in human development. The benefits these bacteria confer to the host can explain why they account for the predominant proportion of the microbiota. *Bacteroides* comprise one of the largest genera in phylum Bacteroidetes, and are responsible for production of succinate and acetate [34], whereas the Firmicutes genera *Roseburia* and *Faecalibacterium* are butyrate producers [35]. Short-chain fatty acids, including acetate and butyrate, have been shown to improve gut barrier function [36], suppress insulin-mediated fat accumulation in adipose tissue [37], and protect the host against colonic diseases [38].

Data from the comparative analysis of gut microbiota among ethnic groups showed no significant differences between groups at the phylum level, though we observed large significant differences at the genus level. In the Tibetan cohort, *Butyricimonas*, *Oscillibacter*, *Barnesiella*, and *Catabacter* were found in greater relative abundance than in the Han group while *Butyricimonas* and *Paraprevotella* were found in significantly lower relative abundance in the Hui group than in the Tibetan group. With the exception of *Oscillibacter* and *Barnesiella*, the relative abundances of all other genera were less than 1%. Previous studies have reported that *Oscillibacter* make potentially substantial contributions to the development of metabolic diseases and gut dysfunction [39,40]. In contrast, some *Barnesiella* species are considered beneficial bacteria in the human gut [41]. For example, Ubeda et al. [42] found that members of genus *Barnesiella* provided resistance to intestinal colonization and blood infection by vancomycin-resistant *Enterococcus faecium* in a mouse model. Thus, we speculate that the high abundance of *Oscillibacter* and *Barnesiella* in the Tibetan group may indicate the presence of beneficial taxa.

Dietary intake is also an important factor affecting the composition of gut microbiota. Food components that are indigestible for human enzymes provide substrates for intestinal microbial metabolism [32]. In the present study, dietary structure was not significantly different across ethnic groups, although consumption of fruits, seafood, vegetables, and poultry meat were significantly different. We also found that 17 genera were correlated with diet. Previous studies reported that *Bacteroides* was positively associated with diets rich in animal fat and protein, whereas the *Prevotella* was associated with low intake of fats and proteins and high intake of carbohydrates and simple sugars [43,44]. However, we found no relationship between the major food sources and the abundance of *Bacteroides* and *Prevotella*, most likely owing to the weakness of the correlation.

Grains, vegetables, and fruits are the main sources of dietary carbohydrates, which can be fermented by the gut microbiota when they are not absorbed in the upper gastrointestinal tract [35]. Our results showed that mixed grains, which consisted of rice, wheat, and maize, were positively correlated with *Catabacter*, *Morganella*, and *Dehalococcoides*. Similarly, correlations between gut microbiota and grains have also been identified in other studies. Zhou et al. [45] fed mice with whole grain oat or low bran oat flour for eight weeks and found that the two experimental groups had different compositions of intestinal microbiota. Carvalho-Wells et al. [46] found that maize-derived whole grain was correlated with an increased level of bifidobacteria in the human gut microbiota. Although different types of grains have varying effects on gut microbiota composition, a relationship clearly exists between grain intake and gut microbiota.

In addition to correlations between grains and individual constituents of the gut, we further discovered that *Clostridium*_*IV* was positively correlated with milk intake, and *Clostridium*_*XlVa* was positively correlated with fruit intake. *Clostridiales cluster XlVa* and *Clostridiales cluster IV* are normally the two most abundant groups of human fecal bacteria that produce butyrate [47]. Moreover, *Clostridium* species are reported to increase the expression of regulatory T cells, which regulate the immune response in the large intestine, and may help to maintain the gut barrier function [48]. High fruit and milk intake may thus contribute to the high relative abundance of *Clostridium*_*IV* and *Clostridium*_*XlVa* in the gut microbiota. *Bifidobacterium* abundance was positively correlated with nut intake. *Bifidobacterium* is a probiotic that performs many physiological functions, such as assimilation of dietary fibers, modulation of mucosal immunity, and regulation of gut flora via competitive exclusion of pathogenic bacteria, resulting in decreased pathogen colonization [49,50,51]. We thus found that diet provides a strong influence in selecting for specific taxa in the gut community.

In light of several studies showing large differences in the microbial communities purportedly due to differences in ethnicity (that is, differences in genetic and cultural background), we sought to refine the extent of this impact by controlling for factors associated with geographic separation of communities, such as pollution or particulate matter in air or bacterial inocula in water or regional food supplies. To address this issue, we selected school-age children from three distinct ethnic groups, all in the same town and socio-economic status, who presumably share exposure to the same environmental reservoirs of potential colonists. A previous study found significant differences in community structure between Hadza and Italian individuals who had different dietary habits using PCoA of weighted and unweighted UniFrac distances [29]. Li et al. [52] found clear differences in beta diversity (Jaccard or Bray-Curtis distances) among three Tibetan groups from traditional, semi-urban, and urban herdsmen, originating from three adjacent areas with different dietary regimens.

Surprisingly, the PCoA conducted in our study did not show distinct clustering patterns among the three ethnic groups. We propose that ethnicity may have less of a role in shaping gut microbiota than previously suggested, given the following factors: (1) all participants recruited in this study were from the Labrang Primary School and resided in Xiahe County of the Gannan Tibetan Autonomous Prefecture which had similar environment and economic conditions; (2) although the school children came from different nationalities, they had similar dietary habits, except for fruits, vegetables, seafood, and poultry meat, which were significantly different among the three groups; (3) the proportions of sex, BMI, and age were not significantly different among the various groups. Thus, we confirmed that ethnic differences had no impact on the beta diversity of gut microbiota under similar geographical and dietary factors. However, the alpha diversity (observed species, ACE, and Shannon indices) showed significant differences among the three ethnic groups, which indicated that ethnicity may play a role in regulating the alpha diversity of gut microbiota. In agreement with our findings, principal coordinate analysis by Deschasaux et al. [14] revealed no clear separation of groups by beta diversity, although the alpha diversity of gut microbiota differed among five ethnic groups, consisting of 2084 participants, all living in the same city.

## 5. Conclusions

To our knowledge, this study represents the first investigation of the isolated effects of ethnicity and diet on gut microbiota composition and diversity in school-age children. In contrast with other studies showing a strong impact on diversity related to ethnic background, our results demonstrate that in the absence of geographic distance and environmental variation, ethnicity significantly contributes to differences in the diversity and predominant taxa within groups (alpha diversity), as determined by observed species, ACE, and Shannon indices, but does not significantly contribute to beta diversity. These findings indicate that genetic and cultural differences are likely overestimated as a driving force in microbial diversity, when in fact geo-spatial separation and environmental differences may contribute more strongly to divergence in microbiota. Diet also affects microbial diversity, although in these intermingled communities the variation in diet and nutrition between ethnic groups was not wide enough to significantly affect diversity. This work provides valuable data on the composition of microbiota in school children from different genetic and cultural backgrounds, and insight into the relationship between ethnicity and microbial diversity, so that these similarities can eventually be exploited in the treatment of digestive disorders.

## Figures and Tables

**Table 1:** Average daily food intake among the three ethnic groups.

Food Species	Han (*n* = 50)	Tibetan (*n* = 44)	Hui (*n* = 49)
	Intake (g)	Intake (g)	Intake (g)
Grains	156.25 (84.13–258.75)	110.88 (69.50–237.75)	115.00 (72.81–212.75)
Vegetables	151.69 (63.04–305.75) **	87.81 (52.07–253.94)	77.25 (33.61–121.55)
Fruits	149.27 (74.82–278.94) **	103.67 (44.34–191.98)	73.71 (22.30–158.95)
Poultry meat	16.00 (3.67–37.52)	20.35 (4.91–59.88) *	10.58 (2.80–44.48)
Livestock meat	3.05 (0.73–10.88)	1.59 (0.00–6.26)	4.58 (0.87–16.55)
Seafood	2.78 (0.85–11.71) *	1.67 (0.00–10.24)	0.76 (0.00–4.97)
Milk	94.00 (37.38–202.75)	116.63 (50.00–282.38)	61.75 (16.75–141.75)
Beans	13 (0.00–53.25)	11.75 (0.00–46.25)	2.29 (0.00–20.25)
Eggs	9.47 (0.00–21.20)	11.36 (1.51–35.95)	10.70 (0.81–26.68)
Nuts	9.32 (1.87–28.82)	8.55 (1.38–23.08)	4.36 (1.28–15.40)
Condiments	83.00 (38.75–194.00)	63.13 (33.06–132.81)	48.91 (11.19–291.56)
Sweets	8.00 (1.50–27.38)	10.13 (0.69–30.25)	5.84 (1.68–35.63)

Condiments include salt, vinegar, monosodium glutamate, pepper, soy sauce, and mustard. * compared with the Hui, *p* ˂ 0.05; ** compared with the Hui, *p* ˂ 0.01; *p* value was calculated using Kruskal–Wallis rank sum test.

**Table 2:** Energy and mean percentage of energy from macronutrients among the three ethnic groups.

Nutrient	Han (*n* = 50)	Tibetan (*n* = 44)	Hui (*n* = 49)
Energy (kcal)	1945.12 (743.82–2768.64)	1727.85 (1191.41–2636.43)	1052.77 (818.14–2514.21)
Proteins (% energy)	13.79 (11.12–18.90)	13.04 (11.67–15.27)	14.61 (11.04–17.73)
Fats (% energy)	28.72 (20.61–38.75)	33.93 (28.84–38.83) **	27.49 (17.83–35.95)
Carbohydrates (% energy)	54.66 (47.96–64.18)	51.55 (45.50–56.54)	59.16 (46.63–69.18)

** compared with Hui, *p* ˂ 0.01; *p* value was calculated using Kruskal–Wallis rank sum test.

## Supplementary Materials

Supplementary materials can be found at https://www.mdpi.com/2076-2607/8/2/254/s1. Figure S1. Rarefaction curves representing the observed number of species in the three groups. The y-axis indicates the number of OTUs per sample. The x-axis indicates the number of sequences randomly extracted from the sample. Figure S2. LEfSe analysis of signature taxa in the Han, Tibetan, and Hui groups. (a) Linear discriminant analysis. (b) Cladogram report. Prefixes represent the abbreviations for the taxonomic rank of each taxon: phylum (p_), class (c_), etc. Figure S3. Observed species and Shannon indexes comparing the alpha diversity of diets among ethnic groups. Figure S4. Beta diversity analysis by PCA examining the diversity of food components in the diets of each ethnic group.
