# Supplementary figures and images for: Ethnic Differences Shape the Alpha but Not Beta Diversity of Gut Microbiota from School Children in the Absence of Environmental Differences
Source: Microorganisms. 2020 Feb 14;8(2):254. doi: 10.3390/microorganisms8020254 (PMC7074779; doi:10.3390/microorganisms8020254)

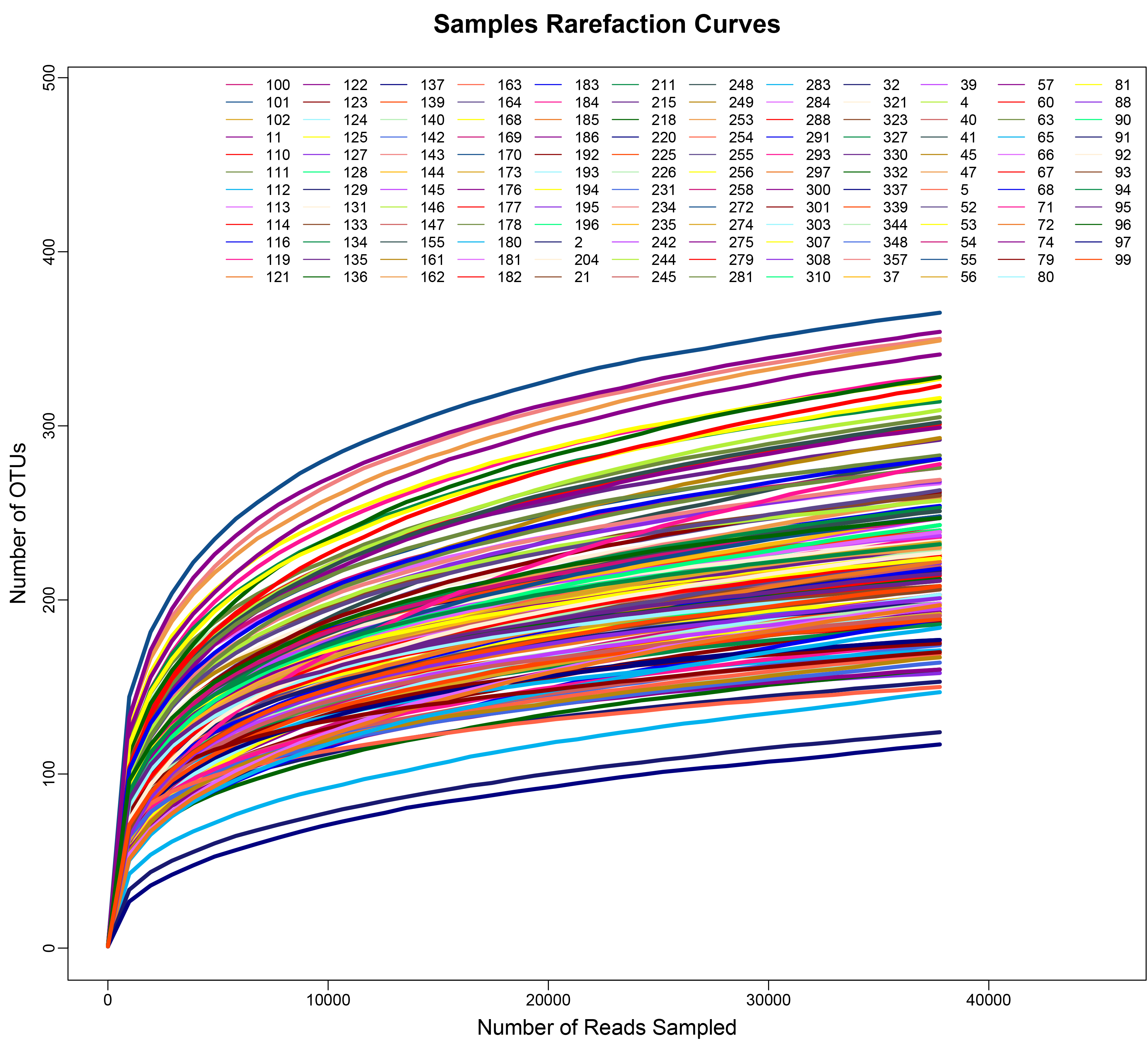

Supplement: Supplementary file 1 [file microorganisms-08-00254-s001.zip › Supplementary material/Figure S1.jpg]

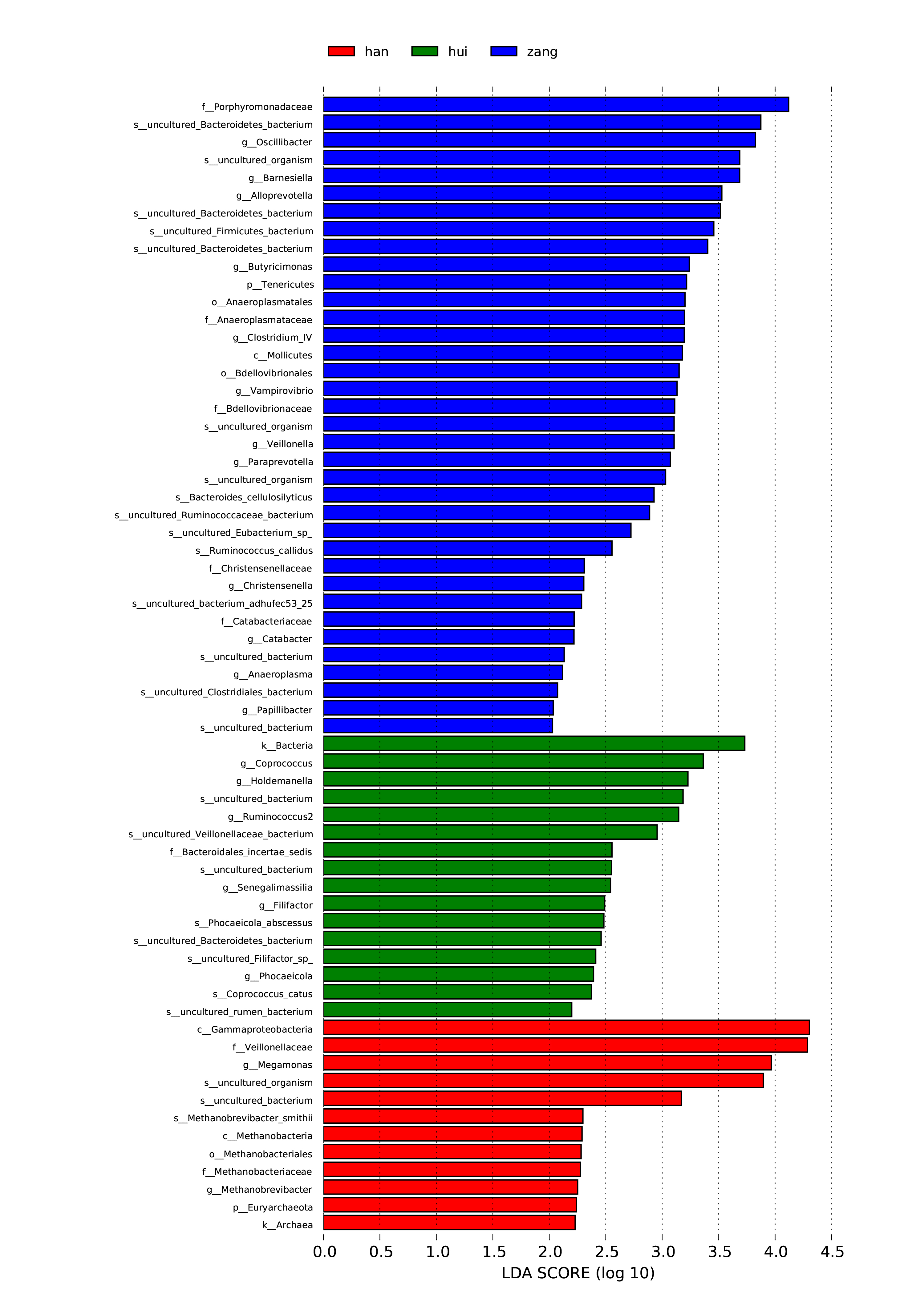

Supplement: Supplementary file 1 [file microorganisms-08-00254-s001.zip › Supplementary material/Figure S2.a.jpg]

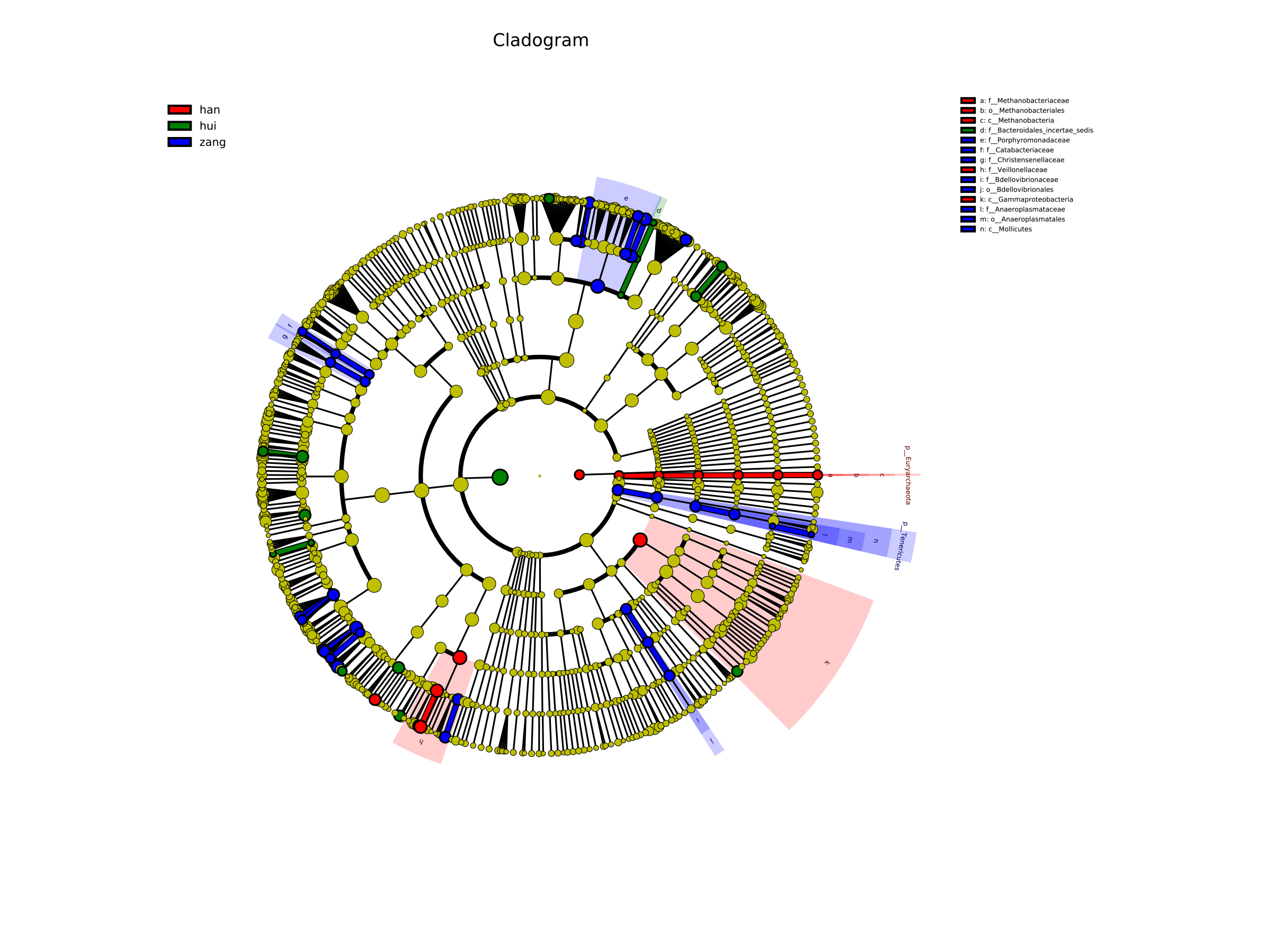

Supplement: Supplementary file 1 [file microorganisms-08-00254-s001.zip › Supplementary material/Figure S2.b.jpg]

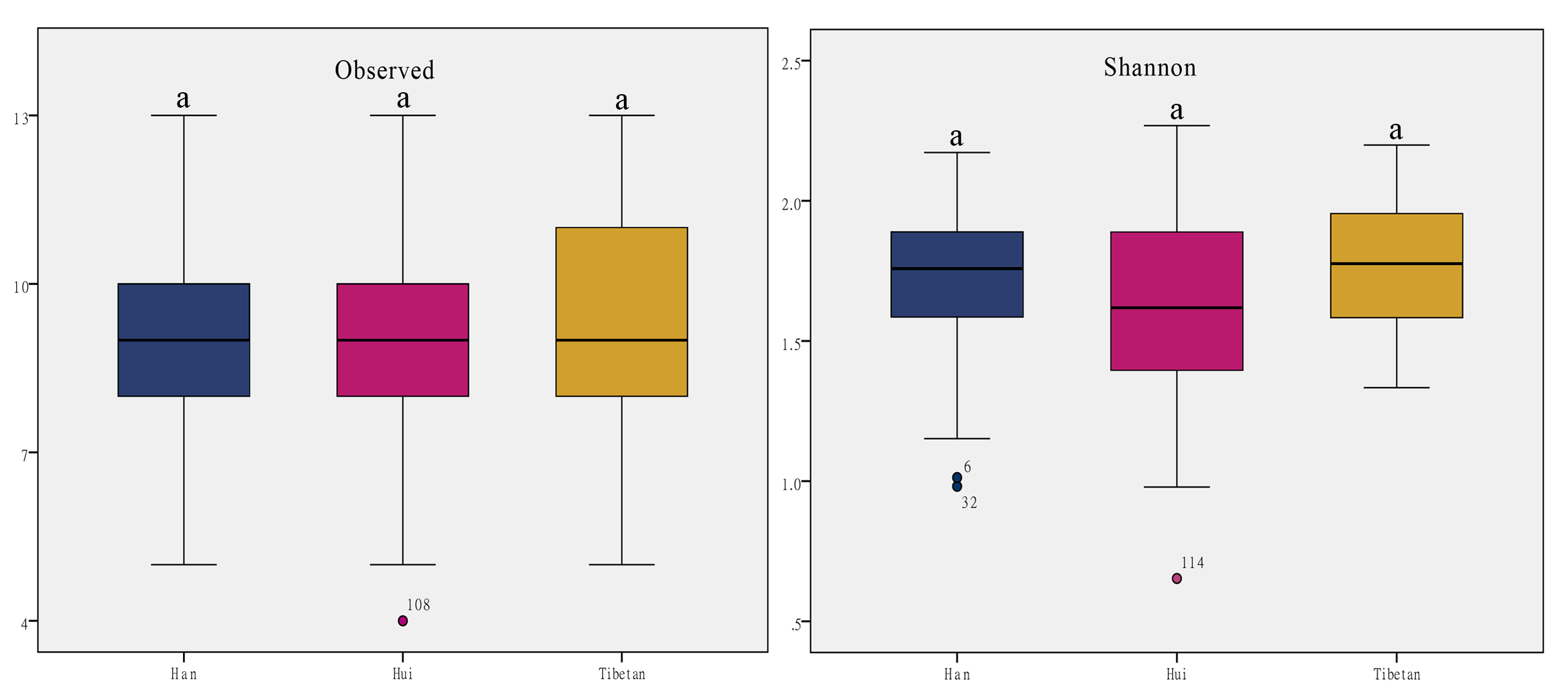

Supplement: Supplementary file 1 [file microorganisms-08-00254-s001.zip › Supplementary material/Figure S3.jpg]

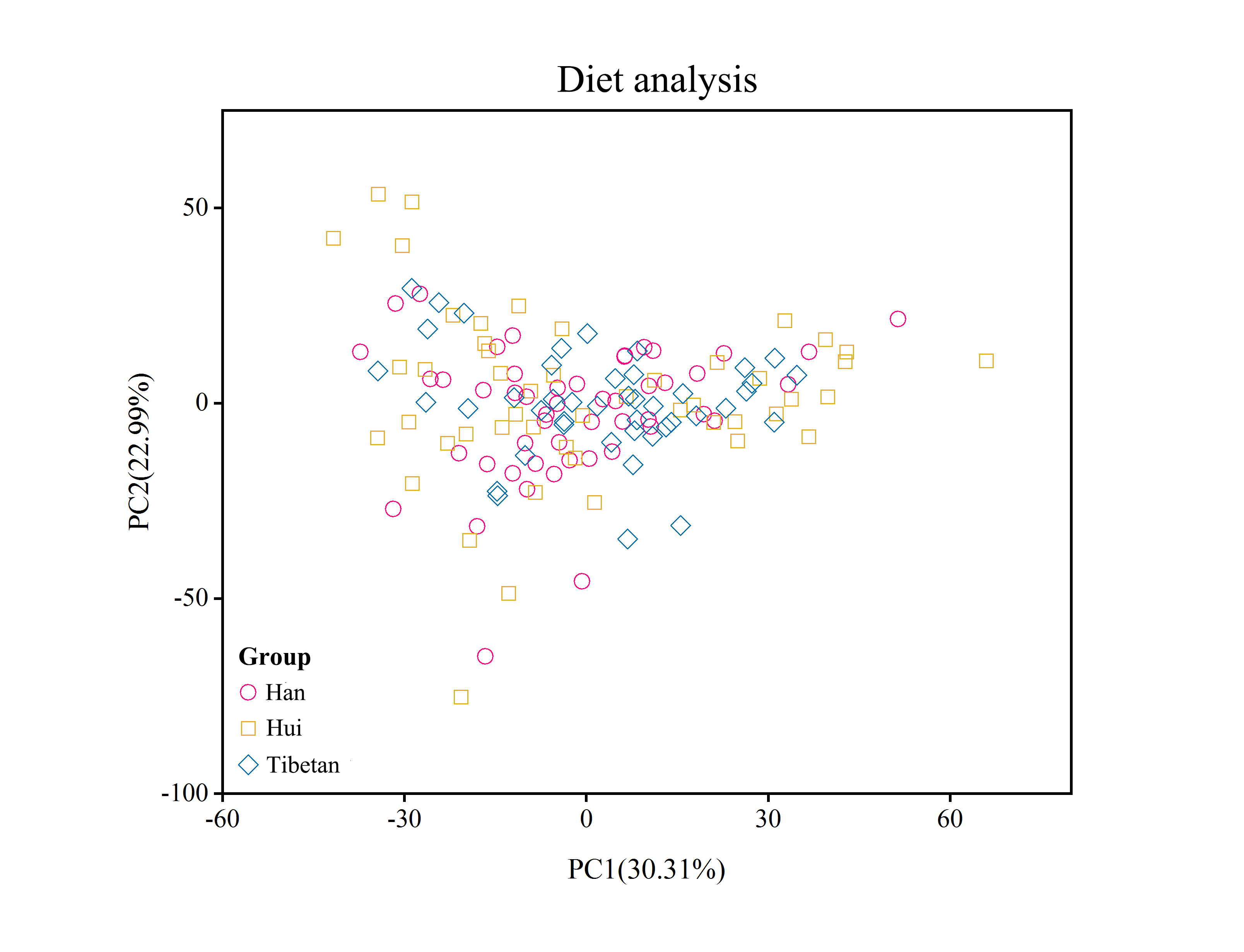

Supplement: Supplementary file 1 [file microorganisms-08-00254-s001.zip › Supplementary material/Figure S4.jpg]
